# Analysis of lncRNA-Mediated ceRNA Crosstalk and Identification of Prognostic Signature in Head and Neck Squamous Cell Carcinoma

**DOI:** 10.3389/fphar.2019.00150

**Published:** 2019-03-04

**Authors:** Yunbao Pan, Guohong Liu, Dujuan Wang, Yirong Li

**Affiliations:** ^1^Department of Laboratory Medicine, Zhongnan Hospital of Wuhan University, Wuhan University, Wuhan, China; ^2^Department of Radiology, Zhongnan Hospital of Wuhan University, Wuhan University, Wuhan, China; ^3^Department of Clinical Pathology, Houjie Hospital of Dongguan, The Affiliated Houjie Hospital of Guangdong Medical University, Dongguan, China

**Keywords:** head and neck squamous cell carcinoma, ceRNA network, long non-coding RNAs, microRNA, TCGA

## Abstract

Long non-coding RNA (lncRNA) can act as ceRNA to regulate the expression of target genes by sponging miRNAs, and therefore plays an essential role in tumor initiation and progression. However, functional roles and regulatory mechanisms of lncRNAs as ceRNAs in head and neck squamous cell carcinoma (HNSCC) remain to be determined. We downloaded RNA sequence profiles from The Cancer Genome Atlas (TCGA) database, and identified the differential RNAs by bioinformatics. Then we analyzed the biological processes of differential expressed RNAs (DER), and established their interaction networks and pathway analysis to find out potential biological effects of these DERs. Besides, we also explored the relationship between the DERs and prognosis of HNSCC patients. We obtained 525 tumor samples and 44 paracancerous controls, and there were 1081 DElncRNAs, 1889 DEmRNAs, and 145 DEmiRNAs. GO and KEGG pathways analysis of these DEmRNAs were mainly involved in “Protein digestion and absorption,” “Calcium signaling pathway,” and “ECM-receptor interaction.” The analysis of the ceRNA network identified 61 DElncRNAs as functional ceRNAs whose dysregulated expression may affect the expression of oncogenes/tumor suppressor genes. Furthermore, univariate and multivariate Cox regression analysis revealed that 4 DElncRNAs, 3 EDmiRNAs, and 6 DEmRNAs can predict survival with high accuracy. Survival analysis found that 4 lncRNAs was related to prognostic, including overexpressed RP11-366H4.1, HOTTIP, RP11-865I6.2, and RP11-275N1.1 patients had a worse survival. In conclusion, through constructing the ceRNA network in HNSCC patients, we identified key lncRNA-miRNA-mRNA network in HNSCC. All the DERs might participate in varieties of pathways in the initiation, progression, and invasion of HNSCC. Furthermore, some miRNAs (hsa-mir-99a, hsa-mir-337, and hsa-mir-137) and mRNAs (NOSTRIN, TIMP4, GRB14, HOXB9, CELSR3, and ADGRD2) may be the prognostic genes of HNSCC. This study provided a new target and theoretical basis for further research on molecular mechanisms and biomarkers.

## Introduction

Head and neck cancer refers to malignant tumors derived from the nasal cavity, paranasal sinuses, nasopharynx, oral cavity, pharynx, and larynx; the majority of these tumors are squamous cell carcinomas and their carcinogenesis has been associated with cigarette smoking (Hashibe et al., 2009), alcoholism (Hashibe et al., 2009) and HPV (Herrero et al., 2003). Head and neck squamous cell carcinoma (HNSCC) is the sixth most common cancer worldwide with an incidence of over 650 000 new cases each year (Torre et al., 2015). Despite recent advances in molecular pathology and targeted therapies have remarkably improved the prognosis of patients with HNSCC, there are no specific biomarkers or comparable effective targeted molecular therapies available to screen and treat HNSCC patients, and the 5-year survival for patients with HNSCC still remains low (Argiris et al., 2008). There is an urgent requirement for the investigation of prognostic biomarkers and treatment options based on the current genomic approaches for HNSCC.

The development of high-throughput sequencing endowed a powerful tool to expand the understanding of gene expression mechanisms. It has been demonstrated that non-coding RNAs play important roles in regulating gene expression (Wang and Chang, 2011). Non-coding RNAs can be divided into microRNA (miRNA, 20–23 nucleotides) and long non-coding RNA (lncRNA, >200 nucleotides) according to the transcript size (Derrien et al., 2012). Plenty of studies have discovered that miRNAs can post-transcriptionally inhibit translation or repress mRNAs expression through binding to the 3′-untranslated region (3′-UTR) of the target genes (Chan and Wang, 2015), leading to cleavage of target mRNAs and/or inhibition of their translation. Currently, it is widely accepted that miRNAs may act as oncogenes or suppressor genes during tumor initiation and progression.

In addition to the well-annotated protein-coding genes and miRNA genes, lncRNAs have also emerged as important regulatory molecules of a variety of physiological and pathological process (Kopp and Mendell, 2018). Studies identified that lncRNA post-transcriptionally regulates the expression of mRNA by competing with miRNA (Feng et al., 2018). Interference of dysregulated lncRNA and mRNAs influence the expression of target mRNAs through miRNAs. This miRNA-regulated lncRNA and mRNA network is a part of the “competing endogenous RNA (ceRNA) hypothesis” (Thomson and Dinger, 2016). The ceRNA crosstalk described that ceRNAs, as miRNA sponges, harbored the same miRNA response elements (MREs) and communicated with each other by competing for shared miRNAs (Thomson and Dinger, 2016). It has proved that lncRNAs could disturb the balance of ceRNA network, thus resulting in the initiation and progress of cancers (Ding et al., 2018; Zhang et al., 2018).

In the current study, to identify the aberrant expression profile of ncRNAs in HNSCC patients and further study the roles of lncRNAs in HNSCC progress, genome-wide profile of ncRNAs was screened by using high-throughput sequencing in HNSCC patients. Further, we analyzed and predicted the functions of the aberrant lncRNAs following the lncRNA-miRNA-mRNA network.

## Materials and Methods

### Study Population and RNA-Sequencing Data Processing

RNA-sequencing data of 546 samples with HNSCC were retrieved from the TCGA data portal^1^. The RNA expression data (level 3) and clinical data of 546 HNSCC cases were downloaded from the TCGA data portal. The RNA- and miRNA-sequencing data from the 546 samples which were free to download were derived from the IlluminaHiSeq_RNASeq and IlluminaHiSeq_miRNASeq sequencing platforms. The sequencing data of the 546 samples contained the corresponding RNA-seq and miRNA-seq data and were divided into 502 tumor samples and 44 normal samples. In the current study, we mainly took the program code written in Perl and R language to analyze RNA data.

### Identification of DEIncRNAs, DEmiRNAs, and DEmRNAs

We identified mRNAs and lncRNAs by using the Ensembl database^2^. Before conducting differential expression analysis, we ruled out all unexpressed RNAs by removing all rows with a mean read of less than or equal to one. We analyzed the DEIncRNAs, DEmiRNAs, and DEmRNAs by using the using “edgeR” package, a bioconductor package via R language procedure. All *P*-values used false discovery rate (FDR) to correct the statistical significance of the multiple test. |FoldChange| ≧2 and FDR < 0.01 were considered significant. For the obtained DEIncRNAs, DEmiRNAs, and DEmRNAs, we generated heat maps using the heatmap packages in the R software.

### Construction of ceRNA Network

RNAs sharing the same miRNA regulators affects each other by competing for the limited number of miRNA sponges based on the ceRNA hypothesis. The construction of the ceRNA network included: (a) We obtained the DElncRNAs, DEmiRNA, and DEmRNAs based on above method. (b) The experimentally validated miRNA-lncRNA interactions were downloaded from miRcode database^3^ and then the DElncRNAs and DEmiRNAs were matched with them to obtain their connections, an lncRNA-miRNA regulatory network. Expression correlation (pearson) between DElncRNAs and miRNAs was assessed. (c) Human miRNAs and their targets data were collected as miRNA-gene pairs concurrenced based on the miRDB^4^, miRTarBase^5^, and TargetScan^6^. (d) These two networks, lncRNA-miRNA, and a miRNA-mRNA network were integrated into a comprehensive ceRNA regulatory network to demonstrate the interactions of DEmiRNAs with DElncRNAs or DEmRNAs. The ceRNA network was visualized using Cytoscape v3.4.0^7^.

### Functional Enrichment Analysis

Functional enrichment analysis among GO and KEGG pathway levels was performed based on DAVID Bioinformatics Resources (^8^, version 6.8) and KOBAS 3.0 software^9^.

### Construction of the Prognostic Risk Score

DE genes were assessed by univariate Cox proportional hazards regression analysis to obtain prognostic values (*P* < 0.001), and the most prominent 10 genes were then used to perform a multivariate Cox proportional hazards regression analysis. A risk score formula was established by including each of the prognosis related genes, weighted by their estimated regression coefficients in the multivariable Cox regression analysis. The risk score for each patient was assessed, and patients were classified into high-risk score or low-risk score groups by using the corresponding median risk score as the cut-off. A receiver operating characteristic (ROC) curve was obtained by using R with survival ROC package. Survival difference between the low-risk and high risk group were calculated by the Kaplan Meier and log-rank test.

### Survival Analysis

We further study prognostic DERNAs signature by combining the clinical data of those HNSCC patients. Kaplan Meier curve analysis was performed to evaluate the univariate survival analysis. *P* < 0.05 was regarded as significant unless specifically indicated.

## Results

### Patient Characteristics

The detailed clinical and pathological characteristics of patients were summarized in Table 1. The exclusion criteria were samples without complete data for further analysis. Finally, for RNA-Sequence data (level 3) we matched a total of 501 tumor tissues and 43 paracancerous controls while for miRNA-Sequence data from the same cohort we matched 525 tumor tissues and 44 paracancerous controls.

**Table 1:** Characteristics of the patients (*n* = 501).

Variable		Number (%)
Age		
	≤60	244 (51.2%)
	>60	256 (48.8%)
	Unknown	1 (0.2%)
Sex		
	Male	368 (73.5%)
	Female	133 (26.5%)
Stage		
	I	25 (5.0%)
	II	71 (14.2%)
	III	78 (15.6%)
	IV	259 (51.7%)
	Unknown	68 (13.6%)
Vital Status		
	Alive	283 (56.5%)
	Dead	218 (53.5%)

### Differentially Expressed (DE) lncRNAs, mRNAs, and miRNAs in HNSCC

**FIGURE 1:**
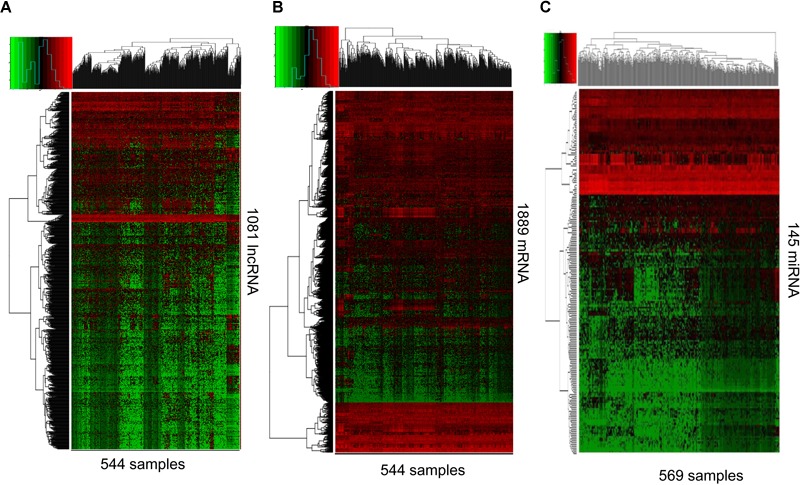
RNA-Seq gene expression profile. Heatmap of 1081 lncRNA **(A)**, 1889 mRNA **(B)**, and 145 miRNAs **(C)** expression in 544, 544, and 569 samples including 43, 43, and 44 normal samples, respectively. Red and green represent high and low expression, respectively.

RNAs expression profiles of HNSCC patients and corresponding clinical data were downloaded from TCGA database. With the cut-off criteria unified |FoldChange| >2 and FDR < 0.01, obtained from TCGA and finally sorted out 1081 DElncRNAs (759 up-regulated and 322 down-regulated) and 1889 DEmRNAs (726 up-regulated and 1163 down-regulated). To further establish an lncRNA-miRNA-mRNA ceRNA network, we also matched miRNA expression profiles in 569 cases of HNSCC patients among the same cohort. As a result, a total of 145 DEmiRNAs (90 up-regulated and 55 down-regulated) were sorted using the cut-off unified |FoldChange| >1.5 and FDR < 0.05. We outlined DElncRNAs, DEmRNAs, and DEmiRNAs using heatmap in Figure 1.

### Reconstruction of the ceRNA Network in HNSCC

**FIGURE 2:**
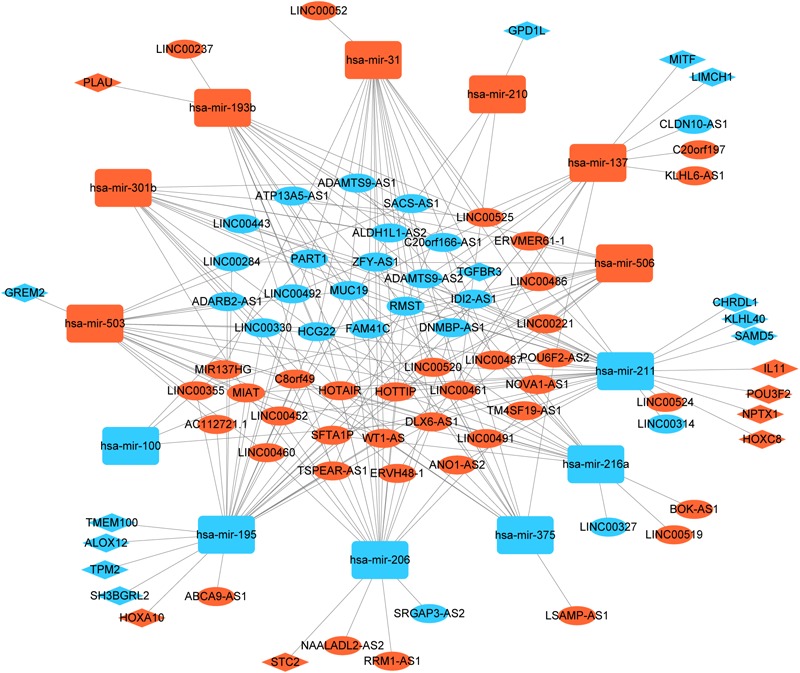
Global view of lncRNA-miRNA-mRNA ceRNA network in HNSCC. The nodes marked in red indicate up-regulated expression while the nodes marked in green indicate down-regulated expression. LncRNAs, mRNAs, and miRNAs are indicated as ellipse quadrangle, and round rectangle, respectively.

To better understand the role of DElncRNAs in HNSCC and to further elucidate the interaction among the DElncRNAs, DEmRNA, and DEmiRNAs, we constructed lncRNA-miRNA-mRNA related ceRNA network. Based on miRcode database, which masters the function of transcriptome-wide mircoRNA targeting prediction including lncRNAs, we outlined 71 DElncRNAs and then we matched above DElncRNAs with previous 145 DEmiRNA, finally, we matched 60 DElncRNAs and 13 DEmiRNAs. Using above 13 DEmiRNAs we predict 500 miRNA-target genes based on these different miRNA-mRNA databases, of which 19 mRNAs were included in the 1888 DEmRNAs. Finally, we sorted out the association between DElncRNAs and DEmiRNAs and the link between DEmiRNAs and DEmRNAs. In the ceRNA network, there were 60 DElncRNAs, 13 DEmiRNAs, and 19 DEmRNAs (Figure 2).

### GO and KEGG Pathway Analysis of DEmRNAs

**FIGURE 3:**
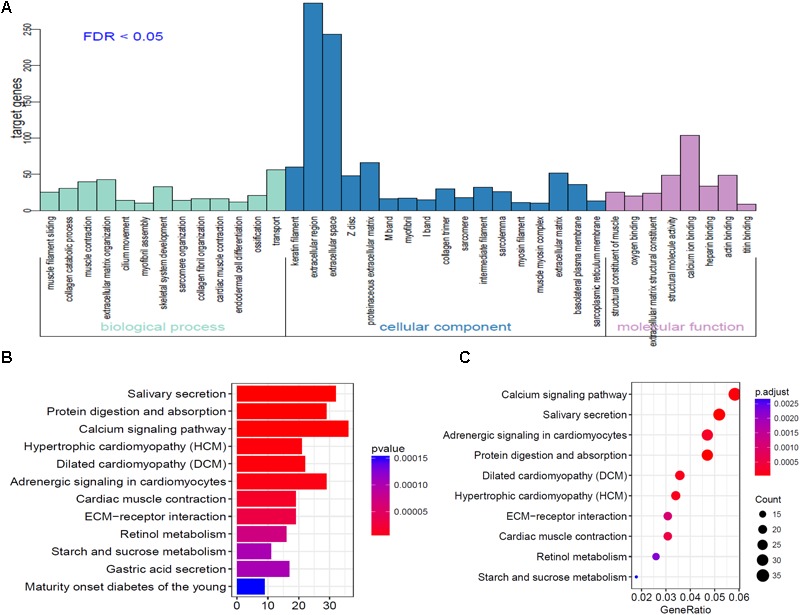
Go analysis using DAVID database of targeted genes in ceRNA network. **(A)** The horizontal axis represents various GO terms, including biological process, cellular component and molecular function. The vertical axis represents the number genes enriched in GO terms. **(B,C)** KEGG pathways based on KOBAS 3.0 website in R.

To further analyze the functional characteristics of DEmRNAsin HNSCC. Gene Ontology (GO) and Kyoto Encyclopedia of Genes and Genomes (KEGG) pathway analysis were performed using DAVID Bioinformatics Tool (version 6.8)^10^ and KOBAS 3.0^11^ websites, respectively. The results were divided into almost three parts, including cellular component, biological process, and molecular function. With the criteria of *P*-Value < 0.01, 38 GO terms were included in Figure 3A. We also analyzed KEGG pathway of the DEmRNAs using KOBAS 3.0. Almost 12 KEGG pathways were significantly enriched in our analysis. The majority of the enriched pathways were associated with cancers including “Protein digestion and absorption, Calcium signaling pathway, ECM-receptor interaction” (Figure 3B,C).

### Survival Evaluation of the DElncRNAs, mRNAs, and miRNAs

**FIGURE 4:**
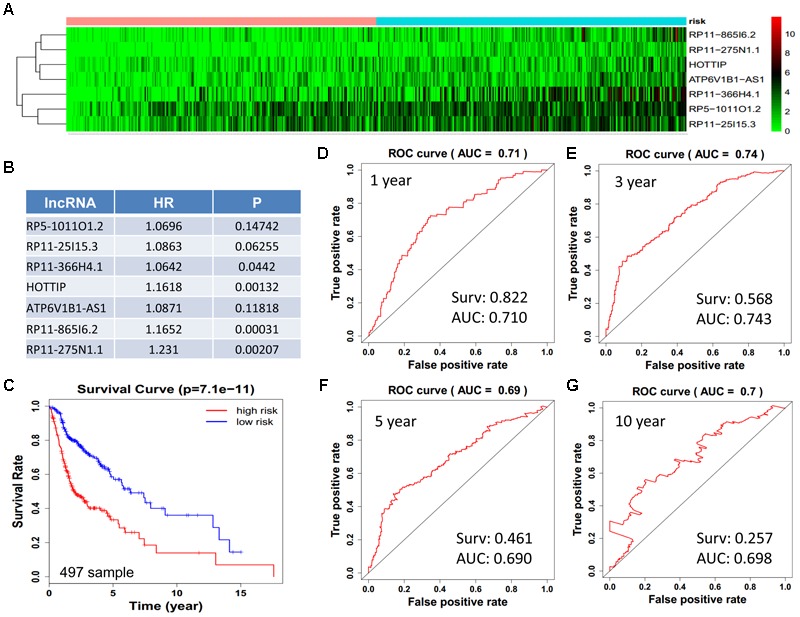
Construction of survival risk score system based on multi-gene signature. Cox regression analysis for survival prediction by the 10-lncRNA signature screened from 22 most significantly expressed lncRNAs with *p* < 1E-3. **(A)** The expression heatmap of the 10 lncRNA in 501 tumor samples; **(B)** the HR and *P*-value in cox model; **(C)** the survival curve of patients with high risk and low risk; **(D–G)** the ROC curve in 1, 3, 5, 10 years with AUC value. Surv: survival rate.

**FIGURE 5:**
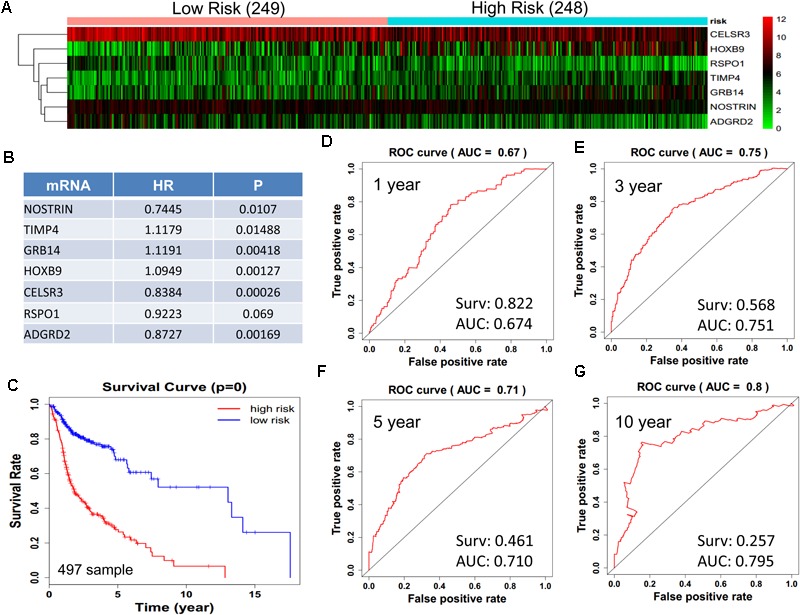
Construction of survival risk score system based on multi-gene signature. Cox regression analysis for survival prediction by the 9-mRNA signature screened from 27 most significantly expressed mRNAs with *p* < 1E-3. **(A)** the expression heatmap of the 10 lncRNA in 497 tumor samples; **(B)** the HR and *P*-value in cox model; **(C)** the survival curve of patients with high risk and low risk; **(D–G)** the ROC curve in 1, 3, 5, 10 years with AUC value. Surv: survival rate.

**FIGURE 6:**
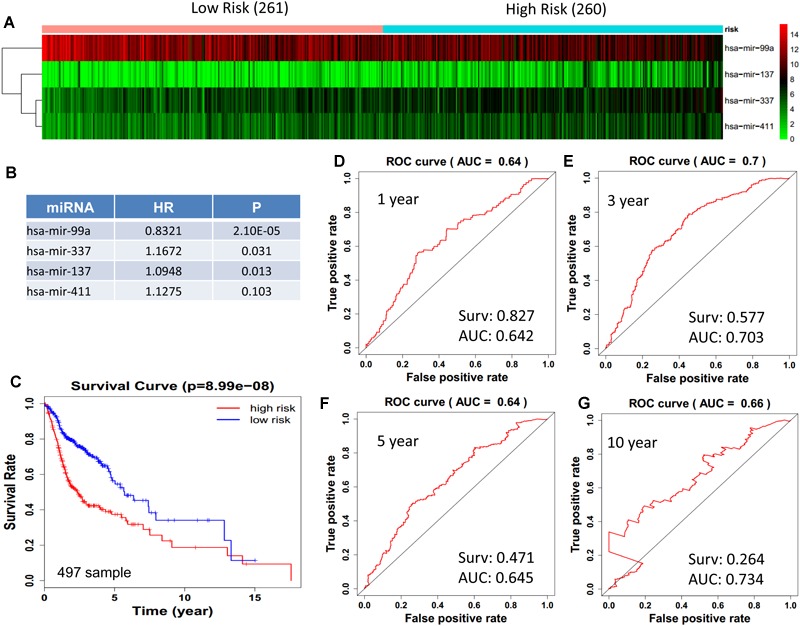
Cox regression analysis for survival prediction by the 13- miRNAs signature screened from 23 most significantly expressed miRNAs with *p* < 0.01. **(A)** the expression heatmap of the 11 miRNAs in 521 tumor samples; **(B)** the HR and *P*-value in cox model; **(C)** the survival curve of patients with high risk and low risk; **(D–G)** the ROC curve for 1, 3, 5, 10 years survival prediction with AUC value. Surv: survival rate.

We obtained 497 cases which had both lncRNA and mRNA expression data and 521 cases which had miRNA expression data for the prognostic evaluation. 496 overlapped cases had lncRNA, mRNA, and miRNA expression data. Using the univariate Cox proportional hazards regression method, 210 lncRNAs, 350 mRNAs, and 41 miRNAs that closely related to the survival of HNSCC with *p*-value < 0.05 were obtained. We select the most closely related 10 lncRNAs, mRNAs, and miRNAs to perform multivariate Cox proportional hazard regression analysis which reduced the amount to be 4 lncRNAs, 6 mRNAs, and 3 miRNAs that can all predict survival as integral with highest accuracy. The expression heatmap of the lncRNA, mRNA, and miRNA signature is shown in Figure 4–6A–C, which is grouped into low risk and high risk groups based on the median value of risk score of each sample. This analysis can predict 1, 3, 5, and 10 years survival rate accurately for HNSCC patients. The time-dependent receiver operating characteristic (ROC) curves for lncRNAs (Figure 4D–G), mRNAs (Figure 5D–G), and miRNAs (Figure 6D–G) signature have area under curve (AUC) values higher than 0.6.

**FIGURE 7:**
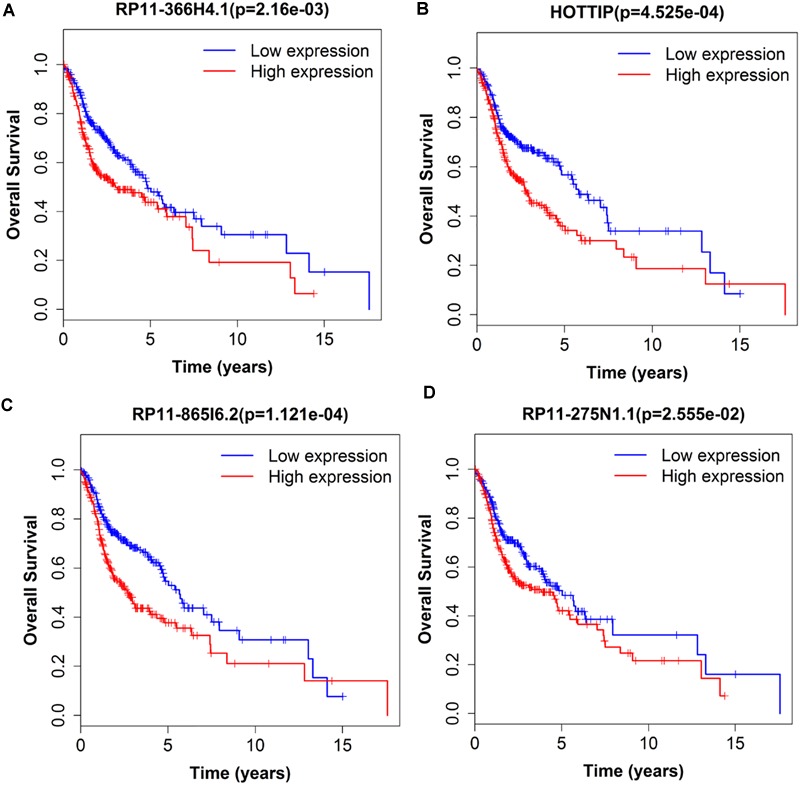
Single lncRNA Survival prediction by the expression level of 4 lncRNAs [RP11-366H4.1 **(A)**, HOTTIP **(B)**, RP11-865I6.2 **(C)**, and RP11-275N1.1 **(D)**] derived from COX analysis which showed significantly differential survival rate. The high and low levels were determined by median value.

**FIGURE 8:**
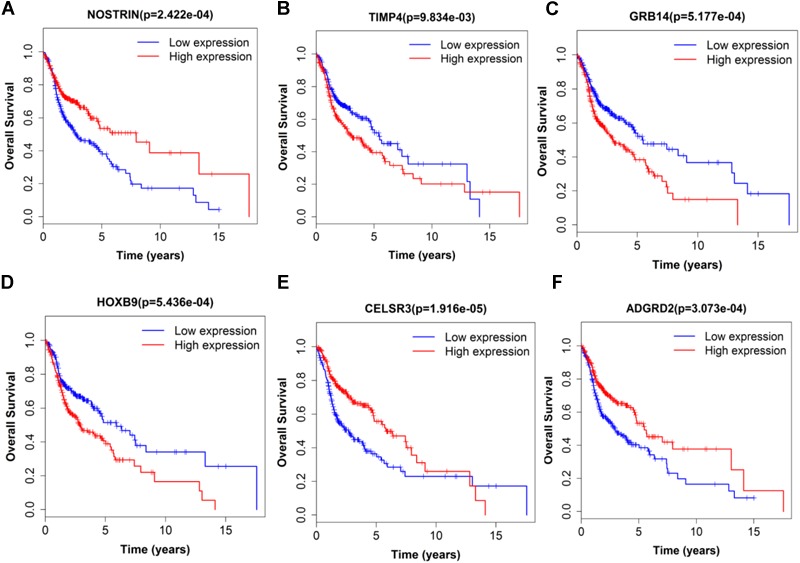
Single mRNA Survival prediction by the expression level of 6 mRNAs [NOSTRIN **(A)**, TIMP4 **(B)**, GRB14 **(C)**, HOXB9 **(D)**, CELSR3 **(E)**, and ADGRD2 **(F)**] derived from COX analysis which showed significantly differential survival rate. The high and low levels were determined by median value.

**FIGURE 9:**
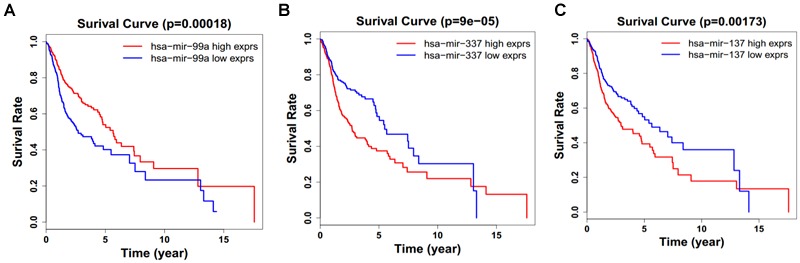
Single miRNA Survival prediction by the expression level of 3 miRNAs [hsa-mir-99a **(A)**, hsa-mir-337 **(B)**, and hsa-mir-337 **(C)**] derived from COX analysis which showed significantly differential survival rate. The high and low levels were determined by median value.

**FIGURE 10:**
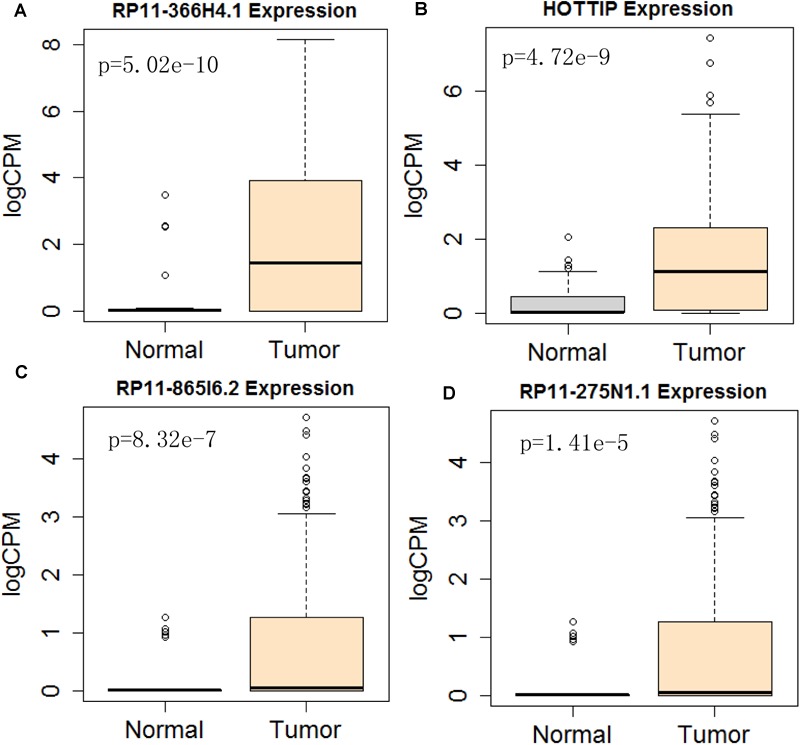
Differential expression profile of the 4 lncRNAs [RP11-366H4.1 **(A)**, HOTTIP **(B)**, RP11-865I6.2 **(C)**, and RP11-275N1.1 **(D)**] derived from COX analysis as compared to normal tissue. The number of normal control and tumor are 43 and 501, respectively.

**FIGURE 11:**
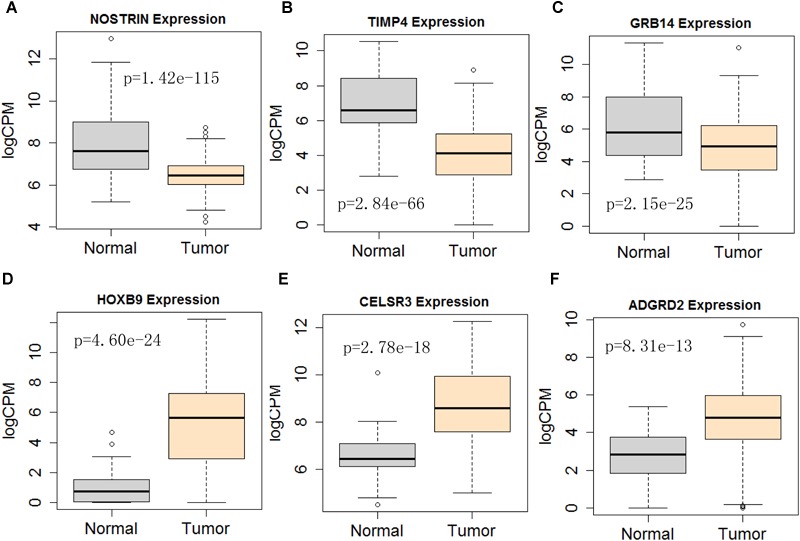
Differential expression profile of the 6 mRNAs derived [NOSTRIN **(A)**, TIMP4 **(B)**, GRB14 **(C)**, HOXB9 **(D)**, CELSR3 **(E)**, and ADGRD2 **(F)**] from COX analysis as compared to normal tissue. The number of normal control and tumor are 43 and 501, respectively.

**FIGURE 12:**
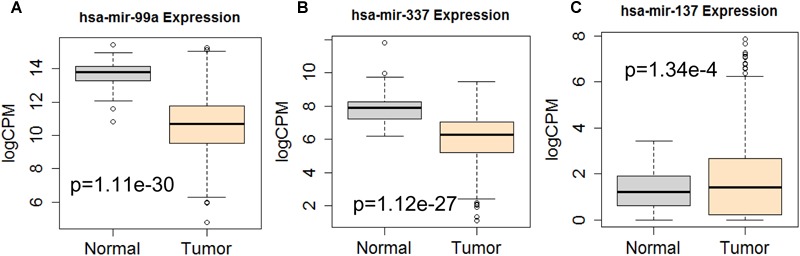
Differential expression profile of the 3 miRNAs [hsa-mir-99a **(A)**, hsa-mir-337 **(B)**, and hsa-mir-137 **(C)**] derived from COX analysis as compared to normal tissue. The number of normal control and tumor are 44 and 522, respectively.

Next, we performed single gene survival analysis from the above 4-lncRNA, 6-mRNA, and 3-miRNA to see if these genes can act independently as prognostic biomarker, based on low and high expression group which is divided by the median value of gene expression level. Among these genes, 4 lncRNAs, 6 mRNAs, and 3 miRNAs showed significant differential survival between high expression and low expression samples with *p*-value < 0.05 which is shown in Figure 7–9. The expression profile of the 4 lncRNAs, 6 mRNAs, and 3 miRNAs in tumor sample all showed significant differential expression as compared to normal tissue samples and are shown in Figure 10–12, respectively.

## Discussion

The prediction of HNSCC prognosis largely depends on the TNM staging system and histologic grade (Alsaffar et al., 2016). It is very important to investigate novel prognostic biomarkers for HNSCC.

Benefit from the development of sequencing technology, the genome-wide profiling has extensively discovered that almost 98% of the transcriptional outputs are ncRNAs. The recent implication of lncRNAs in many biological functions has established a new scenario to better understand complex processes like cancer (Joung et al., 2017; Bester et al., 2018). Meanwhile, miRNAs have been well investigated in the regulation of transcription, epigenetics modulation, and RNA-protein interaction (Gorbea et al., 2017; Thomou et al., 2017). Indeed, some miRNAs have been proven to be potential diagnostic and therapeutic markers for HNSCC (Koshizuka et al., 2017; Chou et al., 2018). However, the combination of lncRNAs and miRNAs is currently not reported for prognosis. In this study, we developed a cox multivariate regression model with survival-related genes. Afterward, the performance of the lncRNA-miRNA-mRNA based risk score was assessed. Patients in the high risk group significantly had a worse survival than that in the low risk group.

GO analysis and KEGG pathway analysis also suggested that the differential expressed genes were associated with several signal pathways, including extracellular matrix organization, collagen fibril organization, which were associated with the pathogenesis of HNSCC (Kuang et al., 2016; Yang et al., 2017). lncRNAs were recognized to regulate downstream signaling pathways by functioning as ceRNA in tumorigenesis (Chen et al., 2018; Li et al., 2018). In our work, we successfully predicted lncRNA/miRNA/mRNA interactions, which may shed a light to reveal the information for ceRNA research in HNSCC.

Accumulative evidence shows that ncRNAs play a very important regulatory role in tumor progression of HNSCC. For instance, lncRNA HOX transcript antisense RNA (HOTAIR) was found to modulate the progression of HNSCC (Sun et al., 2018). HNSCC samples showed significantly robust expression/activation of HOTAIR compared with normal squamous epithelium. Overexpression of HOTAIR promoted the growth of xenograft tumors *in vivo* (Sun et al., 2018). In our ceRNA network, the high expression of the HOTAIR and HOTTIP are closely associated with 6 miRNAs (mir-301b, mir-193b, and mir-31 are upregulated, whilst mir-216a, mir-206, and mir-375 are downregulated). The highly homologous miRNA to hsa-mir-301b, hsa-miR-301a-3p, played a role in the emergence and development of laryngeal squamous cell carcinoma by directly regulating the Smad4 (Lu et al., 2015). MiR-193b was over-expressed in HNSCC cell lines. Knockdown of miR-193b in FaDu cancer cells substantially reduced cell proliferation, migration and invasion, along with suppressed tumor formation (Lenarduzzi et al., 2013). HNSCC patients whose tumors expressed high levels of miR-193b experienced a lower disease-free survival compared to patients with low miR-193b expression (Lenarduzzi et al., 2013). The miR-31 was significantly increased in patients with oral carcinoma at all clinical stages, including very small tumors (Liu et al., 2012), miR-31 also facilitate migration and invasion by targeting Numb in HNSCC cells (Chou et al., 2018). miR-206 and miR-375 were down-regulated in HNSCC clinical specimens and cell lines (Nohata et al., 2011; Liu et al., 2017), which is in agreement with our results. Finally, expression level of miR-216-5p in cervical cancer tissues was observably lower than that in corresponding normal tissues (Zhu et al., 2018).

Considering all the identified lncRNAs, HOTTIP’s abnormal expression had the significant impact on the survival of patients (HR = 1.16; *P* = 0.00132), our data was in agreement with an recent meta-analysis that high HOTTIP expression was significantly correlated with poor OS in cancer patients (Fan et al., 2018). Meanwhile, miRNAs (hsa-mir-99a, hsa-mir-337, and hsa-mir-137) and mRNA (NOSTRIN, TIMP4, GRB14, HOXB9, CELSR3, ADGRD2) were showed to be significant independent factors for poor survival in HNSCC. hsa-mir-99a (Hou et al., 2015), hsa-mir-337 (Wang et al., 2013), and hsa-mir-137 (Langevin et al., 2011) have been reported to be associated with the diagnosis, proliferation, metastasis, and prognosis of various cancers, including HNSCC. Ma et al. (2009) revealed that TIMP4 were differentially expressed and could distinguish cancerous and non-cancerous samples. GRB14 (Morzyglod et al., 2017), HOXB9 (Sun et al., 2017), and CELSR3 (Karpathakis et al., 2016), have been proven to be associated with proliferation and prognosis in multiple cancers, including HNSCC. However, NOSTRIN and ADGRD2 were identified for the first time to be associated with prognosis of HNSCC. More experiments not only in cell lines, but also in animal model, are still needed to investigate the underlying regulatory mechanisms of these ncRNAs.

Taken together, our study identified the lncRNAs expression profile in HNSCC. With the construction of a ceRNA crosstalk network, we constructed a perspective to screen lncRNAs that could be involved in HNSCC tumorigenesis. Moreover, the lncRNA-associated ceRNA network will enable us to better understand the pathogenesis of HNSCC and provide novel lncRNAs and miRNAs as candidate prognosis biomarkers or potential therapeutic targets.

## Author Contributions

YP conceived the study and drafted the manuscript. GL acquired, analyzed, and interpreted the data and reviewed the manuscript. YL reviewed the manuscript and supervised the study. DW helped with discussion and comments on the project.

## Conflict of Interest Statement

The authors declare that the research was conducted in the absence of any commercial or financial relationships that could be construed as a potential conflict of interest.

## Footnotes

1 http://cancergenome.nih.gov/

2 http://www.ensembl.org/

3 http://www.mircode.org/

4 http://www.mirdb.org/

5 http://mirtarbase.mbc.nctu.edu.tw/

6 http://www.targetscan.org/vert_71/

7 http://www.cytoscape.org/

8 http://david.abcc.ncifcrf.gov/

9 http://kobas.cbi.pku.edu.cn/index.php

10 https://david.ncifcrf.gov/

11 http://kobas.cbi.pku.edu.cn/index.php
